# The outcome of open elbow arthrolysis: comparison of four different approaches based on one hundred cases

**DOI:** 10.1007/s00264-013-2172-2

**Published:** 2013-12-03

**Authors:** Maciej Bręborowicz, Przemysław Lubiatowski, Jan Długosz, Piotr Ogrodowicz, Marcin Wojtaszek, Ewa Lisiewicz, Adam Zygmunt, Leszek Romanowski

**Affiliations:** Department of Traumatology, Orthopaedics and Hand Surgery, Poznan University of Medical Sciences, 28 Czerwca 2956r. No.135/147, Poznan, Poland

**Keywords:** Elbow, Stiff elbow, Elbow contracture, Arthrolysis, Surgical approach, Elbow injury

## Abstract

**Purpose:**

The aim of this study was to evaluate the results of elbow arthrolysis according to the surgical approach, durability after arthrolysis and the severity of contracture.

**Methods:**

The study includes a cohort of 100 consecutive patients treated in our institution between 1986 and 2008. The indication for surgery was loss of mobility. This was the result of fractures, dislocation, simultaneous fracture/dislocation or other non-traumatic causes. All patients underwent open elbow release via one of four approaches (42 lateral, 44 medial, six combined medial-lateral and eight posterior). They were clinically evaluated at a minimum of 24 months after arthrolysis.

**Results:**

The average ranges of elbow extension, flexion and arc of motion had increased significantly at the follow up, respectively, by 20°, 16° and 36°. No significant difference was found with regard to surgical approach. However, we noticed significant deterioration of intra-operative average extension and arc of motion (AOM) over the follow up period, respectively, by 13° and 14°. The number of patients with AOM of 100° or more increased from three patients preoperatively to 28 postoperatively.

**Conclusions:**

Open elbow arthrolysis is a successful method of treatment of elbow contracture. Results are durable, but there is some postoperative deterioration of extension gained during surgery. We may anticipate that at the final stage we shall obtain an average of 86 % of intra-operative arc of motion. Patients with the most severe contractures have the best gains.

## Introduction

Elbow stiffness is a common consequence of trauma to the elbow. The elbow’s susceptibility for stiffness has been described before and multiple factors have been implicated [1, 2]. When conservative measures fail, a surgical approach might be indicated. The classic indication is loss of functional range of motion (ROM). The functional ROM has been described by Morrey as minimum extension of 30° and flexion up to 130°. In particular cases even minor limitation may affect specific activities. Elbow arthrolysis is a demanding procedure that has proven to be successful. Many approaches have been described [3–8]. Our aim was to evaluate the clinical results of elbow arthrolysis performed in our institution by different surgeons using four different approaches over a period of 22 years. The secondary aim was to evaluate the impact of different factors on the final result (demographic, surgical, time, etc.).

## Materials and methods

**Fig. 1:**
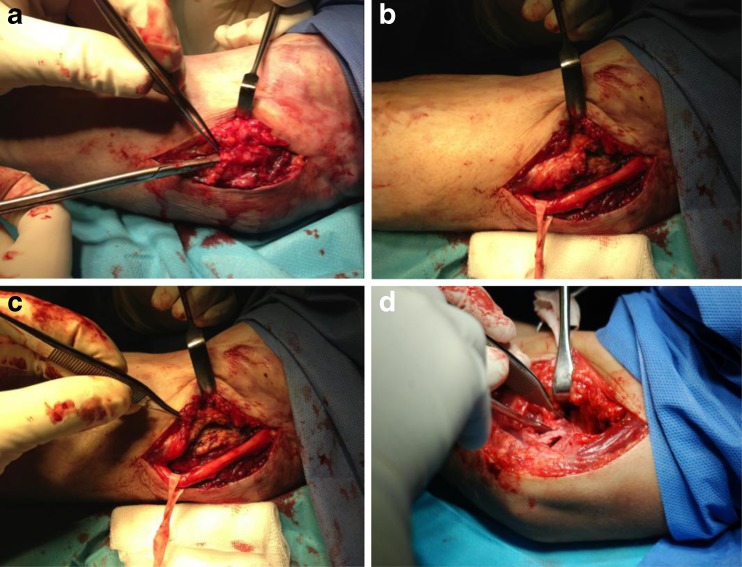
Elbow arthrolysis via the medial approach. The ulnar nerve is exposed (**a**) and protected (**b**), part of the flexor group is released from the medial epicondyle (**c**) and the anterior capsule is released (**d**), then the triceps elevated to access the posterior capsule

**Fig. 2:**
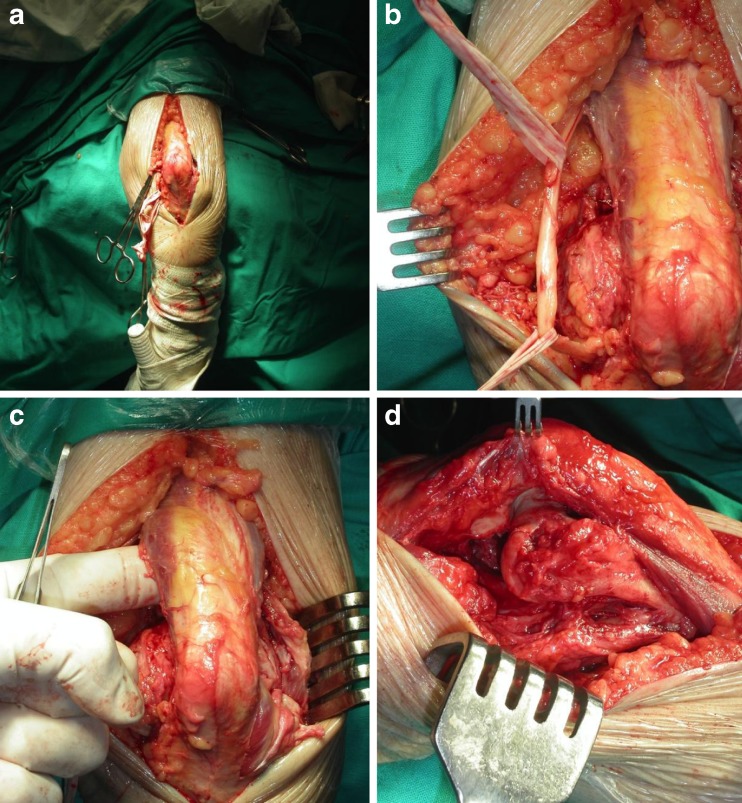
Elbow arthrolysis via the posterior approach. A posterior incision is performed, the ulnar nerve identified (**a**) and protected (**b**), the triceps elevated from the distal humerus and posterior capsule excised (**c**) and the anterior capsule is released (**d**) following detachment of the flexor group from the medial epicondyle

The study included 190 patients operated upon for elbow stiffness in the Department of the Traumatology, Orthopaedics and Hand Surgery, University of Medical Sciences in Poznan between 1986 and 2008, with a minimum two-year follow-up. One-hundred patients agreed to participate in follow-up evaluation. There were 32 women and 68 men. The average age at the time of the procedure was 30 years (range, 2–65). The average follow-up time was 60 months (24–227). All patients underwent open elbow arthrolysis, via lateral approach in 44, medial in 42 (Fig. 1), the combined medial-lateral in six and posterior in eight cases (Fig. 2). The left elbow was treated in 48 patients and the right in 52.

The indication for arthrolysis was the loss of ROM, which resulted from fractures in 58 cases, dislocations in 15 cases, fracture/dislocations in 15 cases and in 12 cases of osteoarthritis. All patients underwent extensive rehabilitation starting immediately after the procedure.

Evaluation was based on the analysis of preoperative and operative data (medical records, history, examination, operating notes) and postoperative follow-up evaluation. Range of elbow motion (flexion and extension) was measured at those three time points using a goniometer. Contracture severity has been classified according to Morrey et al. (Table 1) [9].

**Table 1:** The classification of severity of elbow contracture according to Morrey et al. [9]

Arc of motion	Type of contracture according to Morrey
0–30	Very severe
31–60	Severe
61–90	Moderate
91 and more	Minimal

Statistical analysis was performed using the Statistica® software (version 10, Stat Soft, Inc.). Depending on the type of data that was analysed, it included Wilcoxon test, ANOVA Friedmann test with post hoc analysis, and ANOVA Kruskall–Wallis test with post hoc analysis. Correlations were analysed with Spearman’s test. The study had University Ethical Committee approval and all patients had consented to participate (Nr 1146).

## Results

### General results

**Fig. 3:**
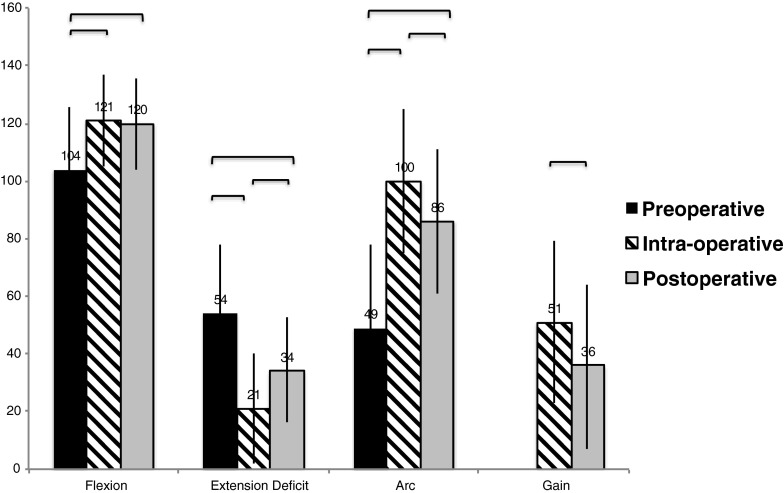
Values of preoperative, intra-operative and follow-up active ranges of motion and the gains. Horizontal brackets—statistically significant *p* ≤ 0.05

The average values of elbow extension, flexion and arc of motion have increased significantly, respectively by 33°, 18° and 51°, immediately at completion of the procedure. Increase in motion remained significant at final follow-up, although it was less than the immediate result with the respective values of 20°, 16° and 36° (Fig. 3). The number of patients with functional ROM (flexion ≥130°, extension ≤30°) increased from two to 14, and the number of cases with an almost functional ROM (flexion ≥120°, extension ≤40°) increased from nine to 38.

There were significant positive correlations between intraoperative and postoperative flexion, extension and AOM values. Moreover, when the gains of values mentioned above had been evaluated, significant positive correlations appeared between the intra versus preoperative gain and post versus preoperative gain.

The gender, age of patients and duration of follow-up time did not influence the final results.

The treatment failed in patients with minimal contractures. There were nine patients showing a postoperative deterioration of amplitude, although all cases had intra-operative improvement of AOM. Among them there was higher percentage of less severe contractures. As compared with the patients with postoperative improvement there were five unchanged contractures (respectively 55 % vs. 1 %), three moderate (respectively 33 % vs. 28 %) and one severe (respectively 11 % vs. 30 %). However, due to the differences of numbers between the groups the statistical analysis was not possible.

### Durability of the results

Significant deterioration of the average extension and arc of motion were observed at the final follow-up (Fig. 3), by 13° and 14° respectively. Patients showed on average 86 % of the AOM obtained during the elbow release.

We observed that extension became significantly lower over time, whereas flexion remained at a similar level.

Interestingly, there was also a group of 23 patients that had improved over time since the procedure. The group was younger, at an average age of 21 as opposed to 32 in the remaining patients. There were also more patients with severe and very severe contracture in that group (respectively 39 % vs. 36 % and 52 % vs. 29 %). They had worse preoperative AOM (by 8°) and intra-operative AOM gain (by 25°).

### Results according to severity of contracture

The greatest improvements of all motion parameters were observed among very severe contractures. Only in cases of minimal contracture were no improvements observed. Functional ROM (AOM ≥100°) had been regained at follow-ups among 14 % of patients with very severe contractures, 21 % with severe, 48 % with moderate and 50 % with minimal contracture. The results are presented in Table 2.

**Table 2:** Severity of preoperative contracture. Results of preoperative, intra-operative and postoperative (at the follow up) extension, flexion and arc of motion (AOM) together with intra and postoperative gain in AOM

Contracture severity		Results			Comparison		
		Preoperative	Intra-operative	Postoperative	Intra vs. preoperative	Post vs. preoperative	Post vs. intra-operative
Very severe (0°–30°) *n* = 36	Extension	71° ± 22°	26° ± 22°	39° ± 16°	*p* ≤ 0.05	*p* ≤ 0.05	*p* ≤ 0.05
	Flexion	90° ± 21°	115° ± 18°	113° ± 14°	*p* ≤ 0.05	*p* ≤ 0.05	N.S.
	Amplitude	19° ± 11°	89° ± 29°	74° ± 23°	*p* ≤ 0.05	*p* ≤ 0.05	N.S.
	Arc gain		70° ± 29°	56° ± 27°			*p* ≤ 0.05
Severe (31°–60°) *n* = 29	Extension	55° ± 19°	24° ± 18°	37° ± 14°	*p* ≤ 0.05	*p* ≤ 0.05	N.S.
	Flexion	104° ± 19°	122° ± 16°	121° ± 14°	*p* ≤ 0.05	*p* ≤ 0.05	N.S.
	Amplitude	48° ± 8°	98° ± 22°	84° ± 20°	*p* ≤ 0.05	*p* ≤ 0.05	N.S.
	Arc gain		49° ± 23°	35° ± 20°			*p* ≤ 0.05
Moderate (61°–90°) *n* = 29	Extension	38° ± 16°	13° ± 10°	27° ± 20°	*p* ≤ 0.05	*p* ≤ 0.05	*p* ≤ 0.05
	Flexion	114° ± 16°	125° ± 11°	126° ± 18°	*p* ≤ 0.05	*p* ≤ 0.05	N.S.
	Amplitude	76° ± 9°	112° ± 17°	99° ± 26°	*p* ≤ 0.05	*p* ≤ 0.05	*p* ≤ 0.05
	Arc gain		36° ± 17°	23° ± 24°			*p* ≤ 0.05
Minimal ( ≥91°) *n* = 6	Extension	28° ± 17°	10° ± 14°	30° ± 21°	N.S.	N.S.	*p* ≤ 0.05
	Flexion	135° ± 9°	136° ± 7°	127° ± 13°	N.S.	N.S.	N.S.
	Amplitude	108° ± 19°	127° ± 15°	97° ± 18°	N.S.	N.S.	*p* ≤ 0.05
	Arc gain		19° ± 14°	−10° ± 10°			*p* ≤ 0.05

*NS* non significant

*p* > 0.05

### Results according to surgical approach

Table 3 shows the results of release related to the surgical approach. The percentage of patients with functional AOM (≥100°) increased in the lateral approach group from 5 % to 34 %, in the medial group from 2 % to 24 % and in the bilateral medial and lateral group from 0 % to 50 %. None of the patients operated upon via the posterior approach obtained postoperative AOM over 100°.

**Table 3:** Surgical approach. Results of preoperative, intra-operative and postoperative (at the follow up) extension, flexion and arc of motion (AOM) together with intra and postoperative gain in AOM

Surgical approaches		Results			Comparison		
		Preoperative	Intra-operative	Postoperative	Intra vs. preoperative	Post vs. preoperative	Post vs. intra-operative
Posterior *n* = 8	Extension	54° ± 30°	30° ± 23°	41° ± 15°	N.S.	N.S.	N.S.
	Flexion	81° ± 34°	116° ± 16°	106° ± 16°	*p* ≤ 0.05	N.S.	N.S.
	Amplitude	28° ± 23°	86° ± 28°	65° ± 12°	*p* ≤ 0.05	N.S.	N.S.
	Arc gain		58° ± 34°	38° ± 29°			N.S.
Medial *n* = 42	Extension	61° ± 25°	26° ± 19°	36° ± 19°	*p* ≤ 0.05	*p* ≤ 0.05	N.S.
	Flexion	106° ± 22°	120° ± 17°	120° ± 15°	*p* ≤ 0.05	*p* ≤ 0.05	N.S.
	Amplitude	45° ± 29°	94° ± 26°	84° ± 23°	*p* ≤ 0.05	*p* ≤ 0.05	N.S.
	Arc gain		49° ± 27°	40° ± 27°			*p* ≤ 0.05
Lateral *n* = 44	Extension	49° ± 20°	16° ± 16°	24° ± 17°	*p* ≤ 0.05	*p* ≤ 0.05	*p* ≤ 0.05
	Flexion	107° ± 19 °	125° ± 14°	122° ± 17°	*p* ≤ 0.05	*p* ≤ 0.05	N.S.
	Amplitude	58° ± 29°	109° ± 22°	88° ± 27°	*p* ≤ 0.05	*p* ≤ 0.05	*p* ≤ 0.05
	Arc gain		51° ± 28°	30° ± 30°			*p* ≤ 0.05
Medial–lateral *n* = 6	Extension	49° ± 36°	13° ± 15°	17° ± 8°	*p* ≤ 0.05	N.S.	N.S.
	Flexion	95° ± 13°	115° ± 23°	119° ± 19°	N.S.	*p* ≤ 0.05	N.S.
	Amplitude	46° ± 24°	103° ± 26°	103° ± 15°	*p* ≤ 0.05	*p* ≤ 0.05	N.S.
	Arc gain		57° ± 37°	57° ± 35°			N.S.

*NS* non significant

*p* > 0.05

We did not find any significant differences when comparing extension, flexion and AOM of patients treated via different approaches preoperatively, intra-operatively and at final evaluation.

## Discussion

The stiff elbow is a major unwanted consequence of trauma. The problem mostly affects the young population and more commonly males. There have been various techniques described in the literature proving successful in regaining functional ROM and improving function [1, 4–8]. There were an almost equal number of cases between very severe, severe and moderate contractures in our study. The approach was chosen according to the surgeon’s discretion, as related to anticipated demand or to previous operations. This research allows comparison of different approaches performed by several surgeons in one institution.

### General results

In most cases the elbow arthrolysis provided significant increase of mobility, with an average gain of 36° (73 %) in the AOM. Functional ROM was difficult to achieve. In our group there were only three patients with AOM of 100° or more preoperatively, while postoperatively the number had increased to 28 (28 %). The average postoperative gains in AOM vary in the literature from 21° to 80° [2, 5, 6, 10]. This diversity may be related to several factors, including operative technique, aetiology, previous experience, time from injury, homogeneity of analysed material and the postoperative protocol [5, 10].

### Durability of results

The influence of time on the final results was assessed by comparing the immediate intra-operative best-achieved ROM with the final result. The average extension and AOM were inferior to the intra-operative values; however, flexion remained stable. A postoperative decrease in the ROM was mentioned in the literature [7–20]; however, significant differences have rarely been reported. Nobuta et al. reported a similar decrease of postoperative extension and lack of significant changes of flexion [12]. The age and gender did not affect the results in our study. The same observation concerning age and gender have been reported in literature [8, 11, 13]. The only exception in our series was the fact that some younger patients had improved intra-operative ROM over time.

The major reason for recurrence of the contracture after surgery or deterioration in ROM can be attributed to the secondary scarring of the joint capsule. Open arthrolysis may be considered as a “controlled injury” to the soft tissues and result in postoperative decrease of ROM [14]. The surgical procedure is a stress factor and causes damage to the tissues, which mobilises inflammatory cells. Substance P and calcitonin-G-related peptide activate mast cells, which release mediators, increasing differentiation and proliferation of the myofibroblasts [15–17]. Transforming growth factor-β positively influences myofibroblast differentiation, while female-sex hormones and TNF-α have negative effects on them [17, 18]. That may be relevant since Hildebrand et al. have reported that the capsule of the stiff elbow contains myofibroblasts in a higher number than the healthy capsule [19]. Interestingly, those cells have the ability to contract the connective tissue via intracellular contractile protein alpha-smooth muscle actin (a-SMA) [16, 20]. There is no clear reason why extension is more prone to deteriorate. However, Germscheid and Hildebrand have shown that the number of myofibroblasts is significantly higher in the anterior capsule of the elbow joint [18].

The process of postoperative scarring might also be the explanation of the results among nine patients who deteriorated postoperatively compared to preoperative values and were operated upon for minimal contractures in our series. For those patients the benefit of release may be overwhelmed by surgical trauma, and possibly less invasive procedures should be considered. All those values qualify preoperative contractures as minimal and moderate. As a result, minimal contractures should be approached by much less invasive arthroscopic techniques.

There was also a group of patients that had improved over time since the operation. It definitely shows that the improvement of the values achieved during the operation among some patients is possible. The young age potential may be one of the explanations. Possible muscular contribution of the contracture that cannot be corrected during surgery might be improved by postoperative rehabilitation. To our knowledge, such observations have not yet been reported.

However, it has to be emphasised that the better the range of movement during operation, the better the final achieved result. We may anticipate that at the final stage we shall obtain an average of 86 % of intra-operative arc of motion.

### Severity

Our patients with a very severe and severe type of contracture showed the greatest improvements in ROM postoperatively. Respectively, the gains of arc of motion were of 294 % and 72 %. As a comparison, the value for moderate contracture was only 30 %. Although the gains among those patients were the greatest, few of them obtained postoperative amplitude of 100° or more. Thus, we can anticipate greater improvement of ROM in more severe contractures, however it will be more difficult to obtain normal or nearly normal elbow function.

There are few papers describing similar findings. Mansat and Morrey have described a similar relationship between the very severe, severe, moderate and minimal contractures and the greatest improvements in the postoperative ROM [6]. Kayalar et al. reported 18 patients with severe contracture out of which 11 could be classified as very severe; the AOM improved from 12.7° to 80° [10].

### The approaches

The surgical approach is probably the most interesting issue from the surgeon’s point of view. There are several factors influencing the choice of approach. It should address the pathology causing stiffness, severity of contracture, presence and location of heterotopic ossification, previous scars from past operations and ulnar neuropathy [12, 21]. In cases where the preoperative flexion is less than 100° some authors suggest that ulnar nerve decompression may be indicated [14, 22].

There are other series that have included patients treated using simultaneous different approaches [11–13], but the authors did not compare them. The choice of the approach was based on the discretion of the surgeon, mostly directed by the possible location of the major reason of contracture. Generally, the results show that all approaches are almost equally effective when proper rules are applied.

Our patients treated with the combined medial and lateral approach had an average AOM gain of 57°. We achieved a better gain in amplitude when compared with the group of Tosum et al. [7] but worse than Kulkarni et al. [23] and Liu et al. [5] who had superior results in AOM gain. The difference in our results and two latter series could be caused by more restricted preoperative motion in those two groups. Kulkarni’s patients had preoperative AOM of 15.6° and Liu et al. had 35°, compared to 46° in our series. Also, in both reports external hinged fixators had been used for better postoperative mobility and rehabilitation. The better gain in AOM can be attributed to this postoperative stabilisation. Unfortunately, the authors do not mention the influence of application of this device on a postoperative change in the ROM, when compared with intra-operative results.

Patients in our series treated with the single lateral approach had worse motion gains when compared with other series [12, 13]. The main difference was the fact that in most of the series CPM was used postoperatively [1, 24]. Also, patients included in other research had formed a more homogenous group than ours—a post-traumatic versus a multi aetiological group respectively.

The gain of AOM among patients being treated with the medial approach was 40°. Compared with the results of the Wada et al. series, a postoperative gain in AOM among our patients was inferior [8]. It is difficult to find the reason for this. The noticeable difference is the fact that in our series we evaluated patients treated with only the medial approach, and in the Wada series four of 14 patients had also been treated by the lateral approach, which was used when medial access was insufficient to release the elbow.

To our best knowledge there are very few English language reports of patients treated only with the posterior approach [25]. However, there are more publications on groups of patients in which some had been treated with the posterior approach. It is therefore hard to compare those series with our results of the posterior approach [13]. Sharma and Rymaszewski reported a series of 25 patients of whom 16 were treated with the posterior approach [13]. Our results concerning the gain in AOM are inferior to theirs, respectively 38° and 55°. This could be explained by the fact that in our series preoperative AOM was inferior by 27° compared to the Sharma and Rymaszewski series, which were respectively 28° and 55°. In our group those patients had the biggest intra-operative gain in AOM by 223 %. Postoperatively the gain decreased to 135 %. In our opinion, the posterior approach is valuable in some cases as mentioned above, concerning both addressing the pathology and final results.

## Conclusions

Open elbow arthrolysis proves to be a successful method, which can be reproduced by different surgeons. It is important to achieve the best possible ROM during the procedure, because that correlates with the final outcome. Results are durable, but some deterioration of extension can be expected over time. Any of the approaches in the study was shown to be effective and the results can be reproduced not only by the expert, but also by many surgeons familiar with similar methods and experience. The choice of approach should address the pathology of the elbow. Patients with the most severe contractures have had the best gain, however their return to normal, or nearly normal ROM, is much less probable.
